# HERV-K Gag RNA and Protein Levels Are Elevated in Malignant Regions of the Prostate in Males with Prostate Cancer

**DOI:** 10.3390/v13030449

**Published:** 2021-03-10

**Authors:** Simin D. Rezaei, Joshua A. Hayward, Sam Norden, John Pedersen, John Mills, Anna C. Hearps, Gilda Tachedjian

**Affiliations:** 1 Life Sciences Discipline, Burnet Institute, 85 Commercial Road, Melbourne, VIC 3004, Australia; sdrezaei@burnet.edu.au (S.D.R.); joshua.hayward@burnet.edu.au (J.A.H.); john.mills@monash.edu (J.M.); anna.hearps@burnet.edu.au (A.C.H.); 2 Department of Microbiology, Monash University, Clayton, VIC 3168, Australia; 3 TissuPath Specialist Pathology, Mount Waverley, VIC 3149, Australia; sam.norden@tissupath.com.au (S.N.); john.pedersen@tissupath.com.au (J.P.); 4 Department of Infectious Diseases, Monash University, Melbourne, VIC 3004, Australia; 5 Department of Microbiology and Immunology at the Peter Doherty Institute for Infection and Immunity, University of Melbourne, Melbourne, VIC 3010, Australia

**Keywords:** human endogenous retrovirus (HERV), prostate cancer, HERV-K

## Abstract

Heightened expression of human endogenous retrovirus (HERV) sequences has been associated with a range of malignancies, including prostate cancer, suggesting that they may serve as useful diagnostic or prognostic cancer biomarkers. We analysed the expression of HERV-K (Gag and Env/Np9 regions), HERV-E 4.1 (Pol and Env regions), HERV-H (Pol) and HERV-W (Gag) sequences in prostate cancer cells lines and normal prostate epithelial cells using qRT-PCR. HERV expression was also analysed in matched malignant and benign prostate tissue samples from men with prostate cancer (*n* = 27, median age 65.2 years (range 47–70)) and compared to prostate cancer-free male controls (*n* = 11). Prostate cancer epithelial cell lines exhibited a signature of HERV RNA overexpression, with all HERVs analysed, except HERV-E Pol, showing heightened expression in at least two, but more commonly all, cell lines analysed. Analysis of primary prostate material indicated increased expression of HERV-E Pol but decreased expression of HERV-E Env in both malignant and benign regions of the prostate in men with prostate cancer as compared to those without. Expression of HERV-K Gag was significantly higher in malignant regions of the prostate in men with prostate cancer as compared to matched benign regions and prostate cancer-free men (*p* < 0.001 for both), with 85.2% of prostate cancers donors showing malignancy-associated upregulation of HERV-K Gag RNA. HERV-K Gag protein was detected in 12/18 (66.7%) malignant tissues using immunohistochemistry, but only 1/18 (5.6%) benign tissue sections. Heightened expression of HERV-K Gag RNA and protein appears to be a sensitive and specific biomarker of prostate malignancy in this cohort of men with prostate carcinoma, supporting its potential utility as a non-invasive, adjunct clinical biomarker.

## 1. Introduction

Prostate cancer is the second most common cancer in males worldwide and the second most common cause of cancer deaths amongst males in many high-income countries [1]. Prostate-specific antigen (PSA)-based screening for prostate cancer was introduced in the late 1980s and has since contributed substantially to early disease detection and the subsequent decline in age-standardised mortality rates [1]. Five-year survival rates for prostate cancer in many settings are now >90%, with some countries achieving early diagnosis in over 80% of cases in either stage I or II [2]. However, one potential consequence of improved detection methods may be the “over-diagnosis” and treatment of clinically insignificant prostate cancers, with a recent analysis suggesting that 42% of prostate cancers in Australia may have been over-diagnosed [3]. A greater understanding of additional, measurable parameters that may facilitate the identification of clinically relevant prostate cancers would be beneficial to prevent physical and psychosocial damages associated with over-diagnosis and treatment.

Human endogenous retrovirus (HERV) sequences constitute 8% of the human genome and represent the remnants of ancestral germline infections by retroviruses previously capable of inter-individual (exogenous) transmission [4]. Although substantial deletions and inactivating mutations have rendered these viruses non-infectious, HERV elements can be transcriptionally active and many sequences retain open reading frames capable of producing viral proteins [5]. Furthermore, HERV regulatory elements located in viral long terminal repeat (LTR) regions can provide tissue-specific enhancers, promoters or alternative polyadenylation signals that may influence the expression of nearby genes [6,7].

There are approximately 40 families of HERVs characterized in the human genome, with the most recently acquired family, HERV-K, retaining the greatest number of intact viral genes [8]. Whilst HERV gene transcription is largely suppressed by epigenetic mechanisms, altered expression of HERV elements is associated with a range of conditions, including neurological diseases, multiple sclerosis, autoimmune conditions, diabetes and, probably most prominently, cancer [3,9]. Enhanced expression of HERV elements through detection of RNA transcripts and viral proteins occurs in a range of cancer types, including breast, melanoma, colorectal and prostate cancers (for recent review see Reference [8]). Malignancy is also associated with increased levels of antibodies to HERV proteins, suggesting that these factors can be immunogenic [10,11] and may represent potential therapeutic targets. Importantly, from a prognostic point of view, a number of studies report associations between heightened HERV activity and disease severity, metastasis and survival [12,13,14]. 

With respect to prostate cancer, previous studies have reported elevated expression of HERV-K and HERV-E RNAs in malignant as compared to normal prostate tissue [15,16,17] and in peripheral blood mononuclear cells (PBMC) of men with prostate cancer [18]. However, substantial variation exists regarding the extent of HERV element upregulation observed in individuals with prostate cancer [15]. This, combined with the observation that factors such as ethnicity and age can affect the expression of HERV mRNA [18], highlights the need to validate HERV expression as a prostate cancer biomarker in different clinical and demographic settings.

In this study, we assessed the expression level of HERV transcripts in matched benign and malignant prostate tissues from men with prostate cancer and compared this expression to prostate cancer-free men. We report evidence of both global increases in HERV transcript expression in prostate tissues of men with prostate cancer as well as heightened expression of HERV-K transcripts specifically in malignant prostate tissue. These findings may be relevant for the development of diagnostic and prognostic biomarkers for prostate cancer.

## 2. Materials and Methods

### 2.1. Study Population and Specimens

Prostate tissue samples were obtained from men diagnosed with prostate cancer (prostatic adenocarcinoma) who underwent radical prostatectomies as part of their treatment. Prostate tissue samples were also obtained from men who underwent radical cystoprostatectomy for bladder cancer, with the prostate tissue subsequently confirmed to be non-malignant. Samples consisted of archival formalin-fixed paraffin-embedded (FFPE) punch biopsies sized 2 × 2 mm (diameter × depth) obtained from men in the greater area of Melbourne, Australia, who had tissues submitted to TissuPath Pathology (Mount Waverley, Australia) for diagnostic pathology. Samples were analysed by a pathologist for the presence of malignant cells and were subsequently defined as being from benign or malignant regions of the prostate. De-identified samples were received for HERV analysis and were analysed blinded. The study was approved by the Alfred Health Human Research and Ethics Committee (Project Number 32/11, approved on 28 March 2011).

### 2.2. Cell Lines and Culture

Primary prostate cancer cell lines DU145, LNCaP, PC3 (HTB-81, CRL-1740 and CRL-1435 respectively, American Type Culture Collection (ATCC)), and the immortalised prostate cell line PNT1A (#95012614, European Collection of Authenticated Cell Cultures), were generously provided by Renee Taylor and Gail Risbridger, Monash University, Clayton, Australia, and cultured at 37 °C in 5% CO_2_ in Roswell Park Memorial Institute media (Gibco, Thermo Fisher Scientific, Waltham, MA, USA) containing 10% foetal bovine serum, 2 mM L-glutamine, 100 U/mL penicillin and 100 µg/mL of streptomycin (all from Thermo Fisher). The immortalised RWPE1 cell line (CRL-11609, ATCC) was cultured in keratinocyte serum-free media supplemented with 5 ng/mL human recombinant epidermal growth factor and 0.05 mg/mL bovine pituitary extract (Thermo Fisher). LNCaP, DU145 and PC3 cell lines are derived from prostate cancer cells and were isolated from a metastatic site (lymph node, brain and bone, respectively), whilst RWPE1 and PNT1A are normal prostate epithelial cells immortalised with HPV-18 and SV40 large T antigen, respectively. 

### 2.3. RNA Extraction and PCR Amplification of HERV Transcripts

RNA was extracted from FFPE tissue using the High Pure FFPE RNA Micro-Isolation Kit (Roche Life Sciences, Penzberg, Germany), with modifications to improve yields including increasing the duration of proteinase K treatment from 30 min to overnight. RNA was extracted from 1 × 10^6^ cultured prostate epithelial cells using the High Pure RNA Isolation Kit (Roche Life Sciences) as per the manufacturer’s recommendations. Purified RNA was treated with RNase-free DNase I (Roche Life Sciences, Penzberg, Germany) to eliminate DNA carryover. cDNA was synthesized using the Transcriptor First-Strand cDNA Synthesis Kit (Roche Life Sciences) as per recommendations using random hexamers and anchored oligo (dT) primers. cDNA prepared from RNA extracted from normal primary human prostate epithelial cells was purchased from ScienCell Research Laboratories (Carlsbad, CA, USA).

The analysed HERV transcripts included four transcripts (HERV-K Gag, HERV-K Np9, HERV-W Gag and HERV-E Pol) previously reported to be upregulated in various cancers, including prostate cancer [19], and two additional transcripts, HERV-H Pol and HERV-E 4.1 surface protein (hereafter referred to as HERV-E Env), were identified through a comprehensive in silico analysis of putative HERV transcripts that possessed an intact LTR promoter, an ATG start codon and an open reading frame, and thus had the potential to express protein. HERV expression was normalised to housekeeping genes glyceraldehyde 3-phosphate dehydrogenase (GAPDH; using forward and reverse primers 5′-TGGTATCGTGGAAGGACTCATGAC-3′ and 5′-ATGCCAGTGAGCTTCCCGTTCAGC-3′, respectively), TATA-box binding protein (TBP; using forward and reverse primers 5′-GAATATAATCCCAAGCGGTTTG-3′ and 5′-ACTTCACATCACAGCTCCCC-3′, respectively) and 18S ribosomal RNA (18S rRNA; Applied Biosystems, Foster City, CA) by qPCR. Table 1 details the location and sequence of primers used to detect these transcripts and the number of amplicons predicted to exist within the reference human genome (hg38). The latter was determined by subjecting each primer pair to a specificity analysis using MFEprimer [20], using the default parameters except for annealing temperature (55 °C), size (900 bp) and reference database (hg38.fa, interim_GRCh38.p10_rna.fa).

cDNA was subjected to PCR using the Brilliant II SYBR Green qPCR Master Mix (Agilent Technologies, Santa Clara, CA) and the following thermocycling parameters on an Mx3000P qPCR machine (Agilent Technologies): initial denaturation step at 95 °C for 10 min, 40 amplification cycles at 95 °C for 30 s and 60 °C for 1 min, followed by melt-curve analysis. The qPCR analysis for all transcripts was performed in triplicate and HERV expression was normalized to housekeeping gene copy number. Plasmid DNA standards were generated for each HERV target by cloning the specific PCR amplicon into a pTOPO cloning vector and amplicon integrity and accuracy were confirmed by DNA sequencing. Serial dilutions of plasmid standards were generated and included in each assay. 

**Table 1:** Details of human endogenous retrovirus (HERV) primers and genomic regions targeted.

Target	Amplicon Size	Primers	Sequence (5′–3′)	Target Region or Reference ^1^	Predicted Targets ^2^
HERV-K Gag	113 bp	Fwd	TTCCCGAGTACGTCTACAGTGA	[19]	1
		Rev	GGTGTTTCTCATCAGGTGGAAT		
HERV-K Np9	113 bp	Fwd	GTTAGTCTACAGGTGTATCCA	GenBank Y17832.2 8836–8948	1
		Rev	CTGTCTCTTTTCCCTACATTTCC		
HERV-W Gag	81 bp	Fwd	TCAGGTCAACAATAGGATGACAACA	[21]	1
		Rev	CAATGAGGGTCTACACTGGGAACT		
HERV-E Pol	98 bp	Fwd	CTCTACACAGTTAGGCTCG	Chr13 40,349,095–40,349,192	1
		Rev	GTGAAAATCCCCGCATAACC		
HERV_E Env	168 bp	Fwd	ATTTGATGCTTGTGCAGCCA	[22]	2 ^3^
		Rev	TTCTTTTTCCAAGTAGCCCAAAT		
HERV_H Pol	118 bp	Fwd	GCCAAACACATATACTCTCC	Chr8 8,066,673–8,066,790	1
		Rev	GAAGAGTGACTGGGATGAAG		

^1^ Chromosome position based on Human Genome Sequence version hg18. ^2^ Number of amplified targets predicted within the genome by MFEPrimer analysis. ^3^ Two DNA targets are on chr17 and chr19, which are both 168 base pairs in length and share 95% nucleotide sequence identity.

### 2.4. Detection of HERV-K Gag Protein by Immunohistochemistry

The presence of HERV-K Gag protein in matched benign and malignant prostate tissue was assessed in FFPE biopsy material from 18 randomly selected participants using immunohistochemistry (IHC). Three micron-tissue sections were cut from selected blocks and stored at 4 °C in desiccated containers until used. IHC was performed using the Leica BOND-Max autostainer (Leica Biosystems, Buffalo Grove, IL, USA). Sections were stained with supernatant from the TI-35 mouse hybridoma raised against HERV-K Gag protein [16] (generously supplied by Prof Eiichi Nakayama, Kawasaki University Medical Welfare, Kurashiki, Japan) for 30 min and detected with the BOND Polymer refine detection kit (Leica Biosystems) with 1/100 dilution and ER2 retrieval for 20 min. Staining was assessed on blinded samples by one of the authors (J.P.), an anatomical pathologist specializing in urological cancers. HERV-K Gag expression was scored individually for benign and malignant sections as either negative or positive, with an overall staining intensity of weak (+), moderate (++), or strong (+++).

## 3. Results

### 3.1. Expression of HERV Transcripts Is Significantly Upregulated in Prostate Cancer Cell Lines as Compared to Normal Epithelial Cells

**Figure 1:**
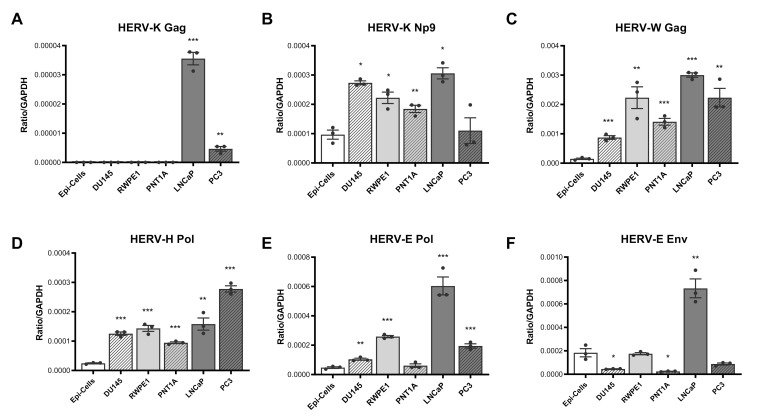
HERV transcript expression in prostate cancer cell lines and primary prostate epithelial cells. Expression of HERV-K Gag (**A**), HERV-K Np9 (**B**), HERV-W Gag (**C**), HERV-H Pol (**D**), HERV-E Pol (**E**) and HERV-E Env (**F**) transcripts were detected in cDNA synthesised from RNA purified from primary non-cancerous prostate epithelial cells (Epi-cells), DU145, RWPE1, PNT1A, LNCaP and PC3 cells by qPCR. Copies of HERV transcripts were standardised to the house-keeping gene GAPDH detected within the same samples. Graphs show the mean and standard error of the mean of results from *n* = 3 independent experiments. *, **, and *** indicate *p* < 0.05, 0.01, and 0.001 respectively, compared to primary epithelial cells, as determined by Student’s unpaired *t*-test.

We determined the expression pattern of six HERV transcripts in cDNA derived from primary prostate epithelial cells and three primary and two immortalised prostate cancer cell lines using qPCR, standardising expression relative to the housekeeping gene GAPDH. This analysis revealed a pattern of over-expression of most HERV transcripts in the majority of prostate cancer cell lines, with some variations. HERV-K Gag expression was significantly and specifically upregulated in LNCap and PC3 cells lines (Figure 1A), whilst HERV-K Np9, HERV-W Gag, HERV-H Pol and HERV-E Pol expression were upregulated in almost all cell lines tested as compared to normal epithelial cells (Figure 1B–E). HERV-E Env expression was more variable, showing a downregulation in DU154 and PNT1A cell lines but an upregulation in LNCaP (Figure 1F). 

To address the possibility that malignant and transformed cells exhibit altered housekeeping gene expression, we also standardised HERV expression to two other housekeeping genes, 18S rRNA and TBP (Supplementary Figure S1). A similar pattern of HERV transcript overexpression was largely observed for HERV-K Gag, -E Pol and -W Gag transcripts, irrespective of the gene used for standardisation. Patterns of expression of HERV-H Pol, -E Env and -K Np9 transcripts relative to normal epithelial cells were also consistent when standardised to TBP, although a different expression profile for these genes was seen when 18S rRNA was used for standardisation. Taken together, these data are consistent with an altered transcriptional state in malignant and transformed prostate epithelial cells, which results in increased expression of a range of HERV transcripts.

### 3.2. HERV-K Gag Expression Is Significantly Upregulated in Malignant, but Not Benign, Regions of Primary Prostate Tissue

To investigate whether expression of HERV transcripts is altered in primary prostate cancer tissue, we analysed matched biopsy samples of both benign and malignant regions of the prostate taken from men diagnosed with prostate cancer (prostatic adenocarcinoma) and compared data with expression patterns observed in prostate biopsies taken from men without prostate malignancy. Prostate tissue samples were obtained from 27 males diagnosed with prostate cancer who had radical prostatectomies, with a median age of 65.2 (range: 47–70). The median Gleason score of prostate cancer donors was 7 (range: 6 to 9), with 2 (7.4%), 18 (66.7%), 4 (14.8%) and 3 (11.1%) donors having Gleason scores of 6, 7, 8 and 9 (out of a possible 10), respectively. The proportion of participants with Gleason score of 8–10 (representing high-grade cancers) in this study was therefore 25.9%. 

Given the variation in housekeeping gene expression observed in prostate epithelial cell lines, we first compared expression of the housekeeping genes GAPDH, 18S rRNA and TBP in the primary tissue biopsies to identify the most robust gene to use for normalisation. The expression of TBP was typically lower than that of GAPDH and 18S rRNA and was undetectable in many biopsy samples. Whilst comparable levels of 18S rRNA and GAPDH were detected per mg of tissue weight, GAPDH was more consistently detected in tissue samples and expression levels did not differ due to malignancy status. Thus, in subsequent analyses, GAPDH was utilised to standardise expression of HERV genes in primary tissue samples.

**Figure 2:**
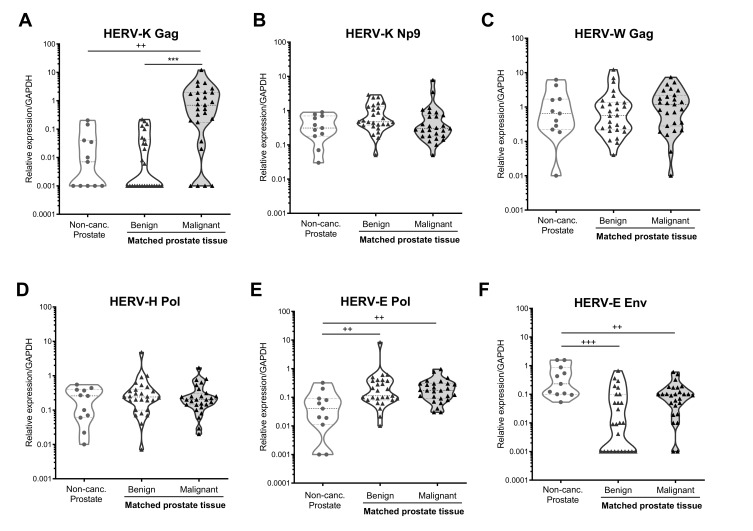
HERV transcript expression in prostate biopsy samples from males with and without prostate cancer. Expression of HERV-K Gag (**A**), HERV-K Np9 (**B**), HERV-W Gag (**C**), HERV-H Pol (**D**), HERV-E Pol (**E**) and HERV-E Env (**F**) transcripts were detected in RNA extracted from punch biopsy prostate samples from men without prostate cancer (Non-Canc. Prostate, *n* = 11), or from matched benign and malignant regions of the prostate from individuals with prostate cancer (*n* = 27) by qRT-PCR. Graphs show truncated violin plots of the average gene expression standardised to copies of GAPDH from *n* = 3 replicates per sample. Samples with undetectable levels of HERV transcript are shown with a value of 0.001 for the purposes of visualisation in the graphs, and the actual value of 0 was used for statistical analyses. ++, and +++ indicate *p* < 0.01 and 0.001 respectively, as compared to non-cancerous prostate tissue, determined by Mann–Whitney U-test. *** indicates *p* < 0.001 for comparison of matched benign and malignant tissue, as determined by Wilcoxon matched-pairs signed-rank test. Non-parametric tests were used due to non-normal distribution of the data.

Analysis of HERV transcripts in primary tissue biopsy samples from prostate cancer-free males indicated that the HERV transcripts examined here were expressed at low but detectable levels in these individuals, with the exception of HERV-K Gag, which was only detected in 55% of samples tested (Figure 2A). Next, we compared HERV transcript expression in these prostate cancer-free individuals with samples taken from benign regions of the prostate in individuals with prostate cancer and found significantly upregulated expression of HERV-E Pol (*p* = 0.003, Figure 2E) and a trend towards increased expression of HERV-K Np9 (*p* = 0.054, Figure 2B), but decreased expression of HERV-E Env (*p* < 0.001, Figure 2F) in men with prostate cancer as compared to those without. The changes to HERV-E Env and Pol expression were also observed in malignant regions of the prostate, suggesting global transcriptional alternations within prostate tissues not specific to the malignant cells themselves. 

Comparison of HERV expression levels between matched benign and malignant regions within the prostate of men with prostate cancer revealed a striking upregulation of HERV-K Gag expression in malignant regions of the prostate (*p* < 0.001, Figure 2A), with 23/27 (85.2%) of donors analysed showing increased HERV-K Gag expression in malignant regions (Supplementary Figure S2). There were no significant differences in expression of any other HERV transcripts analysed between benign and malignant regions of the prostate, although all of the 11 individuals with undetectable HERV-E Env expression in benign samples had detectable expression in malignant regions (Supplementary Figure S2F). Taken together, these data indicate that in this cohort, prostate cancer was associated with global changes in transcription of certain HERV elements in both benign and malignant prostate epithelial cells, and with a significant upregulation of HERV-K Gag expression exclusively in malignant cells.

### 3.3. Analysis of the Relationship between the Expression of Different HERV Transcripts in Malignant Regions of the Prostate

**Figure 3:**
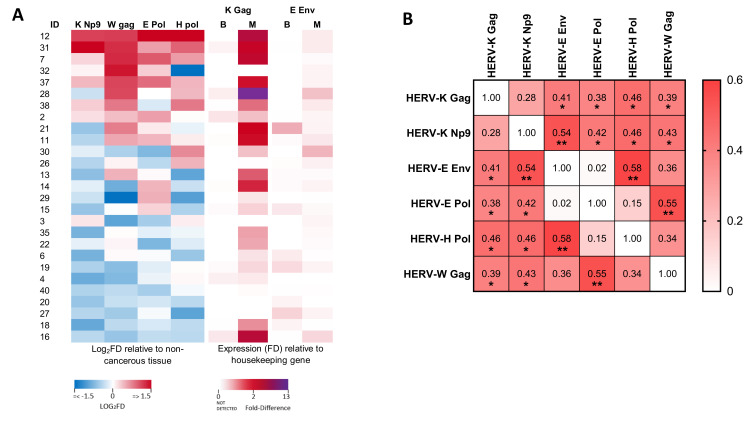
Associations between expression of various HERV transcripts in prostate cancer tissue. (**A**) Heat map showing the differential expression (log_2_ fold difference; FD) of HERV RNA transcripts in prostate cancer tissues from men with prostate cancer. Left panels show expression of HERV-K Np9, HERV-W Gag, HERV-E Pol and HERV-H Pol in malignant regions relative to benign regions of the prostate (each standardised to GAPDH). Right panels show expression of HERV-K Gag and HERV-E Env in both benign (B) and malignant (M) regions relative to expression of GAPDH (due to transcripts being undetectable in many benign tissue samples). (**B**) Correlation matrix showing the association between expression levels of the various HERV transcripts as determined by non-parametric Spearman’s correlation. For HERV-K Gag and HERV-E Env, the expression level in malignant tissues (relative to GAPDH) was analysed, whilst for all other transcripts, the expression in malignant tissue relative to benign tissue (both standardised to GAPDH) was used. Values show Spearman’s rho. * and ** indicate *p* < 0.05 and 0.01, respectively.

Whilst HERV-K Gag was the only transcript analysed which showed significantly altered expression in malignant tissues as compared to matched benign regions at a cohort level, a number of individuals exhibited malignancy-associated alterations to expression of a range of HERV transcripts (Figure 3A). We therefore analysed the association between HERV-K Gag transcript upregulation and other HERV transcripts in men with prostate cancer and found a significant positive association between expression of HERV-K Gag and all other HERV transcripts, except HERV-K Np9 (Figure 3B, Spearman’s rho: 0.39–0.46, *p* < 0.05 for all). Interestingly, HERV-K Np9 expression itself showed a significant positive association with expression of the same transcripts (rho: 0.42–0.54, *p* < 0.05 for all). Significant associations were also observed between expression of HERV-E Env and HERV-H Pol (rho: 0.58, *p* = 0.001), and HERV-E Pol and HERV-W Gag (rho: 0.55, *p* = 0.003). We further investigated whether there was any relationship between HERV expression and age or Gleason score, but did not observe any significant associations. These data are consistent with an altered transcriptional profile in malignant prostate epithelial cells that leads to over-expression of a range of different HERV transcripts, but the magnitude of this upregulation shows substantial inter-individual variation.

### 3.4. HERV-K Gag Protein Is Detected Primarily in Malignant Regions of the Prostate in Men with Prostate Cancer

**Figure 4:**
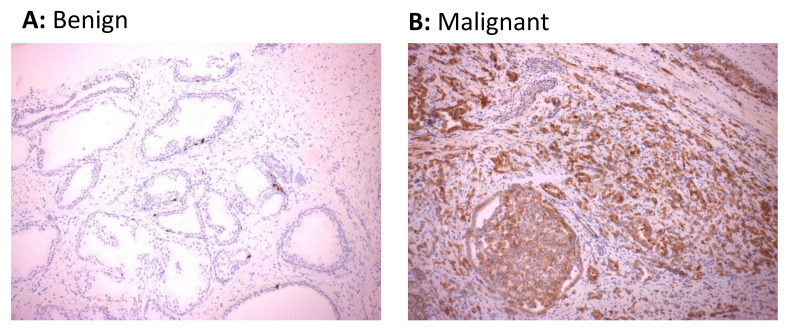
Expression of HERV-K Gag protein in cancerous regions of prostate tissue sections. HERV-K Gag protein was detected in sections of matched malignant and benign regions of formalin-fixed paraffin-embedded prostate biopsy tissue from 18 males with prostate cancer by immunohistochemistry. Immunohistochemical images from benign prostate tissue (**A**) or malignant prostatic adenocarcinoma (**B**) regions of the prostate showing negative and positive staining for HERV-K Gag protein from one representative individual are shown.

Whilst malignancy-associated changes to the transcriptional profile of cells may lead to elevated levels of HERV RNAs, the heavily mutated nature of HERV sequences in the genome may preclude the translation of these sequences into proteins. We therefore performed immunohistochemical staining of biopsy sections from 18 of the 27 study participants to explore whether expression of HERV protein was also upregulated in malignant prostate tissue. For this analysis, we focused on HERV-K Gag given the significant upregulation of this transcript in the majority of participants in this study. Positive immunohistochemical staining of HERV-K Gag protein was detected in 12/18 (66.7%) malignant tissues, with 8 of these 12 positive samples showing areas with medium- to high-intensity staining, but only 1/18 (5.6%) benign tissue sections (Table 2 and Figure 4). Furthermore, comparison of immunohistochemistry results with the extent of transcript expression indicated a trend towards detection of HERV-K Gag protein in samples which showed a greater over-expression of RNA transcript (Table 2). These data indicate that malignancy in prostate epithelial cells is associated with increased transcription and translation of HERV-K Gag sequences and that the detection of HERV-K Gag protein may be a useful discriminatory biomarker of malignancy in this setting.

## 4. Discussion

Malignancy-associated changes in the expression of HERV elements are associated with clinically relevant outcomes in a range of cancer settings and may potentially represent a therapeutic target to specifically identify and target malignant cells. Here, we used matched cancerous and benign tissues from prostate cancer-affected men to demonstrate the specific upregulation of HERV-K Gag RNA and protein within malignant regions of the prostate, highlighting the potential utility of this biomarker to be used for diagnostic, prognostic and therapeutic applications. The finding that heightened expression of HERV-K Gag was restricted to malignant cells suggests that it may be the result of intrinsic cellular changes, such as epigenetic alterations or the action of cell-associated oncogenic factors. We also found significantly elevated expression of HERV-K Gag mRNA in the prostate cancer cell line LNCaP, and to a lesser extent in PC-3 cells, which is consistent with previous findings in some studies [19] but not others [16]. We utilised primers recognising the HERV-K101 provirus but dozens of different HERV-K proviruses have been documented in the human genome with different prostate cancer cell lines showing varied expression of certain loci [23]. This, combined with differences in target amplicons analysed, may influence the expression profiles detected in various studies and highlights that HERV expression profiles are highly sequence- and cell-specific.

Our findings of increased HERV-K Gag mRNA and protein expression in malignant prostate cancerous cells are consistent with the findings of others who showed increased expression of HERV-K Gag mRNA in prostate cancer tissue [15,16] and the detection of HERV-K Gag protein in ≈85% of prostate cancer tissues as compared to approximately ≈40% of normal prostate tissues [15]. Wallace et al. reported elevated levels of HERV-K mRNA not only in prostate tissue from men with prostate cancer but also in PBMC [18], highlighting the potential prognostic utility of this low-invasive parameter. Interestingly, this latter study also identified a significant effect of both race and age on the association between HERV-K element expression and prostate cancer, emphasising the need to validate such biomarkers in the intended target population. Although at a cohort level we did not see a significant difference in expression of HERV-E Env transcripts in malignant as compared to benign prostate tissue, 11 individuals (40.7%) exhibited undetectable levels of HERV-E Env in benign tissue and all of these showed elevated transcript levels in malignant tissue. This is similar to the observations of Wang-Johanning et al. [17], who showed detectable levels of HERV-E Env mRNA in 38.8% of prostate tissues from cancerous patients compared to 5.6% of normal prostate tissue. 

It remains unclear whether the HERV-derived elements play a role in initiating or enhancing malignancy, or whether they are produced as a consequence of epigenetic alterations to cancerous cells. HERV LTRs can act as alternative promoters for host gene expression and modulate expression of genes, including oncogenes and proto-oncogenes (see Reference [24] for a recent review). Whilst the association of HERV overexpression with global DNA hypomethylation in cancer supports the idea that HERV expression is secondary to malignant changes [19], there is evidence that HERV nucleic acids and proteins can potentiate malignancy, with HERV-K Rec and Np9 proteins recognised as proto-oncogenes [25]. Furthermore, HERVs contain an immunosuppressive element which can modify the cytokine response from PBMC, which has been shown in mouse models to induce the recruitment of immunosuppressive cells to the tumour microenvironment [26]. Expression of HERV-H RNA has also been implicated in tumour invasion and metastases both in vitro and in vivo [26], suggesting that in some settings, HERVs may play an active role in potentiating malignant processes. 

Whilst HERV elements have been evaluated as potential targets for adjunct therapies with encouraging results (reviewed in Reference [24]), their most promising role is as diagnostic or prognostic biomarkers of malignancy. In this regard, there is ample evidence linking heightened HERV element expression with clinical outcomes such as metastases, disease severity and mortality in a range of cancer settings, including breast [14,27], melanoma [28] and germ cell tumours [29]. Specifically, in prostate cancers, Reis et al. found that the presence of serum antibodies to HERV-K Gag was almost exclusively restricted to individuals with more severe disease, and those with detectable antibodies tended to have poorer disease outcomes [15]. These findings support a potential role for HERV-K Gag as an adjunct diagnostic marker for prostate cancer, similar to that demonstrated for breast cancer, where HERV-K Gag antibodies and mRNA were found to be a sensitive and specific marker of early-stage cancers [13]. The relationship between expression levels of HERV mRNA, protein and serum antibodies in various malignancies, and which biomarker has the most useful prognostic value, remains to be fully defined.

This study had a number of important limitations, including its relatively small sample size and cross-sectional design, which precluded the analysis of clinical outcomes associated with increased HERV expression. It would therefore be of significant interest to confirm these findings in a longitudinal study of men with a broader range of prostate cancer severities with well-characterised clinical outcomes, to confirm the potential value of HERV over-expression as a useful prognostic marker. Evidence of an association between HERV expression and more severe or progressive prostate cancer may indicate a potential benefit of screening for HERV expression at diagnosis or during ongoing monitoring to inform decisions regarding the need for surgical interventions. Given the ability to detect HERV elements in blood, and the limited but enticing data associating its detection with more severe disease, future work should explore the relationship between HERV expression in tissue and blood compartments to determine whether a minimally invasive blood test could identify cellular changes occurring in prostate tissue. The potential utility of HERV elements as a screening tool or prognostic biomarker to be used in combination with PSA testing to identify high-risk cancers offers the opportunity to further improve prostate cancer prevention and minimise over-treatment of this common cancer.

## Figures and Tables

**Table 2:** Detection of HERV-K Gag protein in malignant and benign prostate tissue from men with prostate cancer.

Sample ID	Age	Gleason Score	HERV K Gag Detected by IHC ^1^		HERV K Gag Detected by qRT-PCR ^4^	
			Benign	Cancerous	Benign	Cancerous
71	59	7	−	+/++	ND	12.3
72	66	7	−	+/++ ^2^	ND	1.2
74	53	7	−	−	ND	ND
75	65	7	−^3^	−	ND	ND
76	62	7	−	+	ND	ND
77	47	8	−	−	0.15	12.1
78	61	6	−^3^	+	ND	0.2
79	69	7	−	++/+++	ND	0.5
80	65	7	−	−	ND	2.7
83	54	7	−	+/+++	ND	ND
84	65	7	−	− ^3^	0.4	1.2
85	66	7	−	+/++	ND	10.1
86	65	8	−	−	ND	0.75
87	69	7	−^3^	+	0.01	4.89
88	70	9	−	++/+++	ND	1.9
94	66	8	+^2^	+	0.02	1.15
97	65	8	−	++/+++	ND	ND
98	64	7	−	++	ND	0.04

^1^ Samples were graded as having no staining (−) or low-, medium- and high-level staining (+, ++ or +++, respectively) by a single experienced Pathologist. ^2^ Variable or focal staining observed. ^3^ Some positive staining in atrophic areas. ^4^ Corresponding expression level of HERV-K Gag mRNA (standardised to GAPDH) for each sample is shown for comparison. IHC: immunohistochemistry, ND: not detected.

## Data Availability

Not applicable.

## Supplementary Materials

The following are available online at https://www.mdpi.com/1999-4915/13/3/449/s1, Figure S1: HERV RNA expression in cell lines and primary prostate epithelial cells, Figure S2: HERV transcript expression in benign and malignant regions of the prostate in men with prostate cancer.
