# Supplementary material for: HERV-K Gag RNA and Protein Levels Are Elevated in Malignant Regions of the Prostate in Males with Prostate Cancer
Source: Viruses. 2021 Mar 10;13(3):449. doi: 10.3390/v13030449 (PMC7999808; doi:10.3390/v13030449)
Supplement: Supplementary file 1 [file viruses-13-00449-s001.pdf]

Supplementary Figure 1

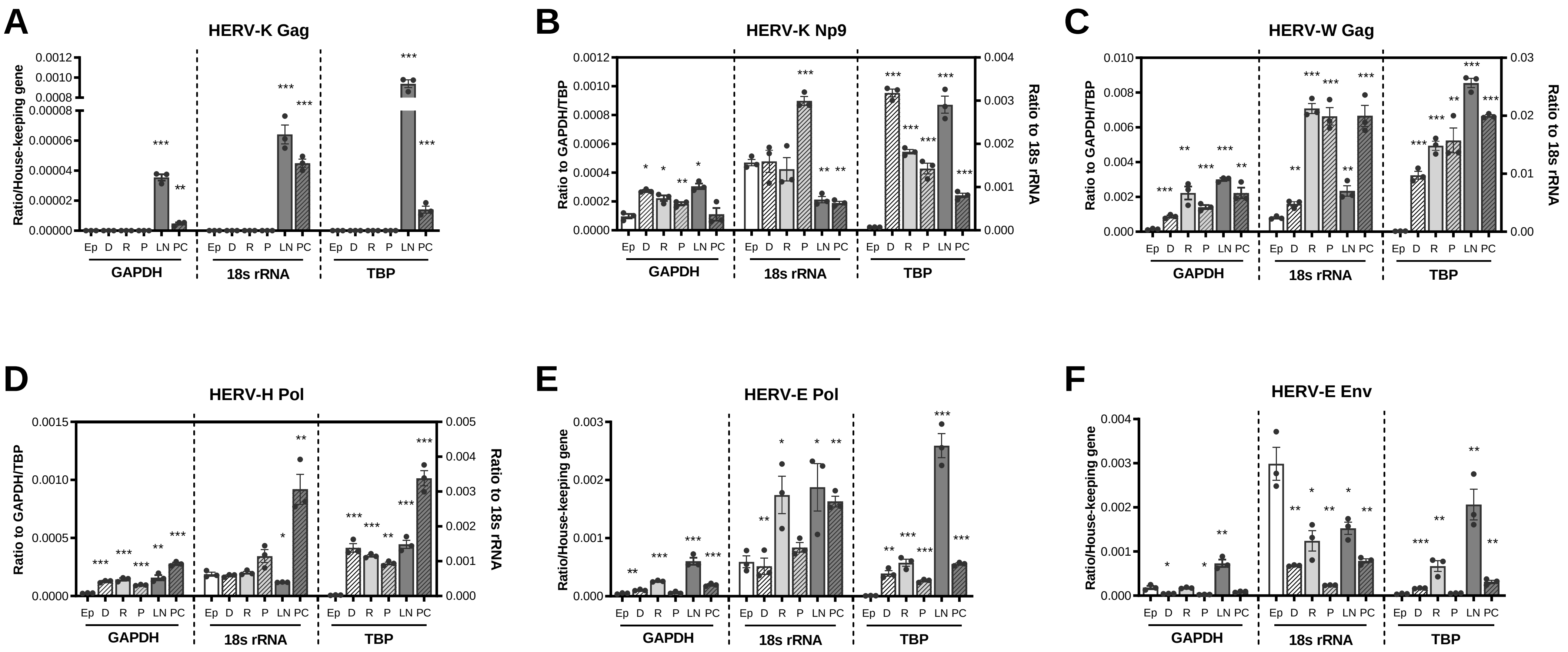

Supplementary Figure 1: *HERV RNA expression in cell lines and primary prostate epithelial cells.*
Expression of HERV-K Gag (A), HERV-K Np9 (B), HERV-W Gag (C), HERV-H Pol (D), HERV-E Pol (E) and HERV-E Env (F) transcripts were detected in cDNA from primary non-cancerous prostate epithelial cells (Ep), DU145 (D), RWPE1 (R), PNT1A (P), LNCaP (LN) and PC3 (PC) cells by qPCR. Copies of HERV transcripts were standardised to the house-keeping genes GAPDH, 18s rRNA and TBP detected within the same samples. In graphs B, C and D the 18s rRNA standardised result is shown on the right y axis. Graphs show the mean and standard error of the mean of results from n = 3 independent experiments. *, **, and *** indicate $p < 0.05$, 0.01, and 0.001 respectively compared to primary epithelial cells, as determined by Student's unpaired t-test.

Supplementary Figure 2

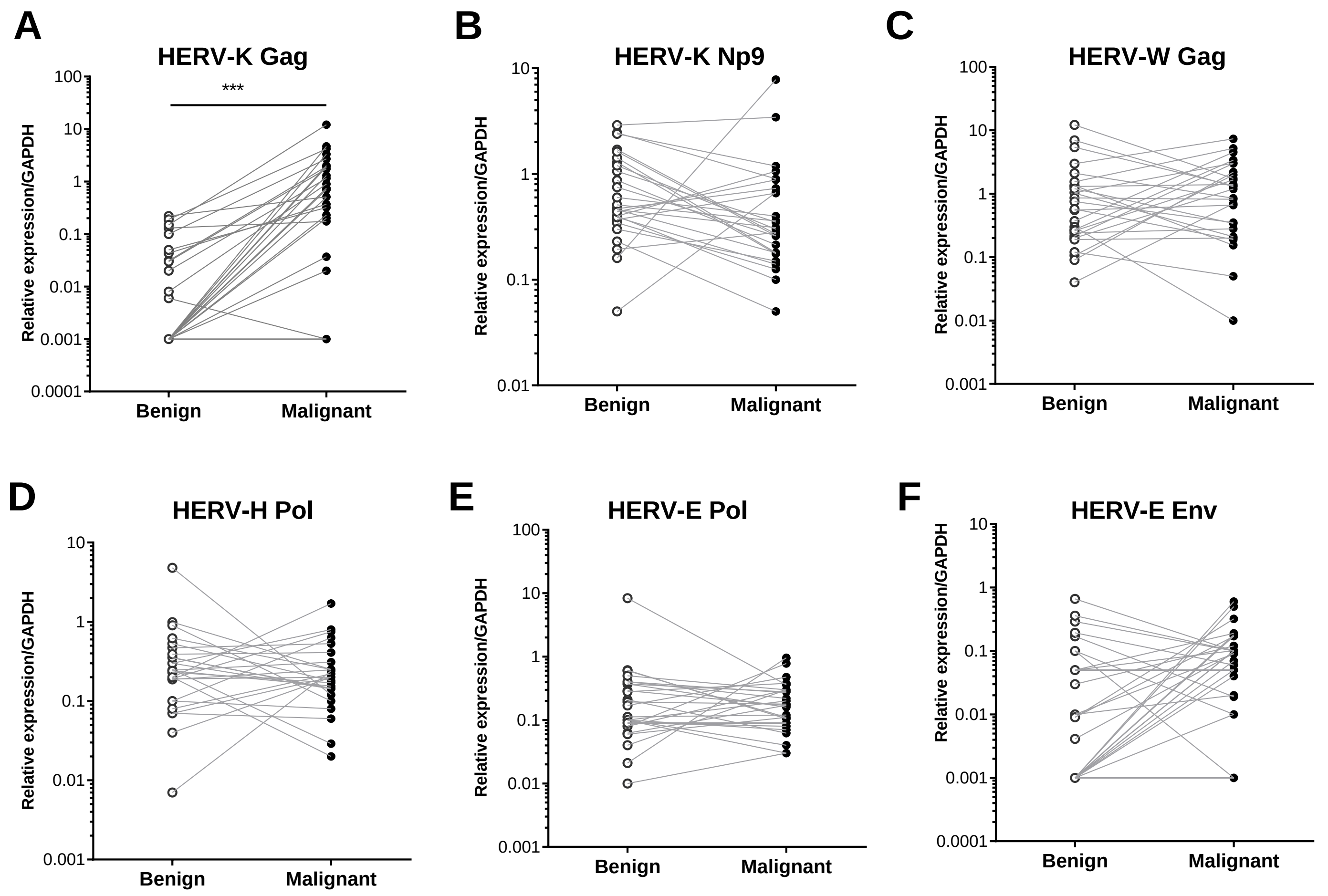

Supplementary Figure 2: *HERV transcript expression in benign and malignant regions of the prostate in men with prostate cancer.* Expression of HERV-K Gag (A), HERV-K Np9 (B), HERV-W Gag (C), HERV-H Pol (D), HERV-E Pol (E) and HERV-E Env (F) transcripts were detected in RNA extracted from matched benign and malignant regions of the prostate from men with prostate cancer (n = 27) by qPCR. Graphs show average gene expression in each donor standardised to copies of GAPDH from n = 3 replicates per sample. Samples with undetectable levels of HERV transcript are shown with a value of 0.001 for the purposes of visualisation in the graphs; the actual value of 0 was used for statistical analyses. *** indicates $p < 0.001$ as determined by Wilcoxon matched-pairs signed rank test.
